# Precise measurement of correlations between frequency coupling and visual task performance

**DOI:** 10.1038/s41598-020-74057-1

**Published:** 2020-10-15

**Authors:** Joseph Young, Valentin Dragoi, Behnaam Aazhang

**Affiliations:** 1 Electrical and Computer Engineering, Rice University, Houston, 77005 USA; 2 Neurobiology and Anatomy, University of Texas John P and Katherine G McGovern Medical School, Houston, 77030 USA

**Keywords:** Computational neuroscience, Visual system

## Abstract

Functional connectivity analyses focused on frequency-domain relationships, i.e. frequency coupling, powerfully reveal neurophysiology. Coherence is commonly used but neural activity does not follow its Gaussian assumption. The recently introduced mutual information in frequency (MIF) technique makes no model assumptions and measures non-Gaussian and nonlinear relationships. We develop a powerful MIF estimator optimized for correlating frequency coupling with task performance and other relevant task phenomena. In light of variance reduction afforded by multitaper spectral estimation, which is critical to precisely measuring such correlations, we propose a multitaper approach for MIF and compare its performance with coherence in simulations. Additionally, multitaper MIF and coherence are computed between macaque visual cortical recordings and their correlation with task performance is analyzed. Our multitaper MIF estimator produces low variance and performs better than all other estimators in simulated correlation analyses. Simulations further suggest that multitaper MIF captures more information than coherence. For the macaque data set, coherence and our new MIF estimator largely agree. Overall, we provide a new way to precisely estimate frequency coupling that sheds light on task performance and helps neuroscientists accurately capture correlations between coupling and task phenomena in general. Additionally, we make an MIF toolbox available for the first time.

## Introduction

The function of different brain regions is often studied by measuring functional connectivity^1^, which considers time-domain or frequency-domain relationships between different neural signals, and correlating such connectivity with task-relevant phenomena such as task performance. In this work, we focus on the frequency-domain aspects of functional connectivity and utilize such aspects to better understand one brain region in particular: the visual cortex. Significant insight into task performance^2^, short-term memory^3^, autism^4^, and epilepsy^5^ has been revealed by frequency-domain relationships, which we refer to as frequency coupling, between neural signals. Although the relationship between frequency coupling and task-relevant phenomena is typically characterized via correlation, frequency coupling itself can be estimated by a variety of different techniques, and our first contribution is the development of a powerful approach for measuring such coupling. Coherence^6^ is perhaps the most common approach and is a frequency-domain version of Pearson’s correlation coefficient. However, coherence is based on second-order statistics, i.e. power spectrums, and therefore is only sufficient when the time series being considered are Gaussian Processes (GPs). Considering that neural signals are almost certainly not Gaussian^5^, full statistical dependence will not be quantified by coherence when used on such signals.

Mutual Information in the Frequency domain (MIF) overcomes this by its data-driven, model-free nature. A recent formulation of MIF^5, 7^ inspired by previous work^8^ showed how mutual information can be applied to the frequency domain to address the aforementioned Gaussian restriction and more fully evaluate frequency coupling. MI in frequency is effectively a generalization of coherence that can evaluate nonlinear relationships between two time series. It does so without any model constraint by quantifying true statistical dependency across frequency components of each time series.

**Figure 1:**
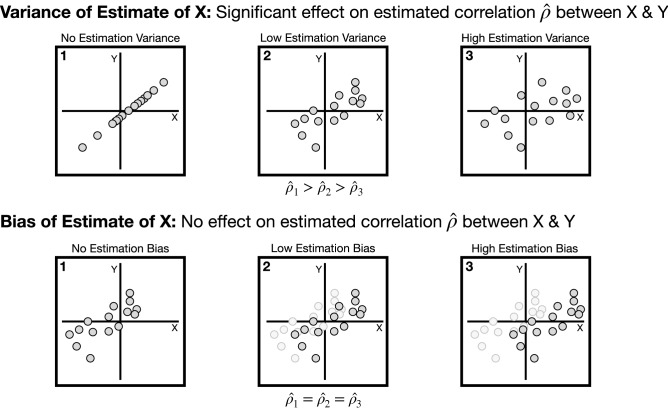
Illustration of how the variance in estimating *X* is inversely related to the maximum measurable Pearson correlation coefficient $\rho (X,Y)=cov(X,Y)/\sigma _X\sigma _Y$^11–15^, while the bias in estimating *X* does not affect the correlation. Therefore, one should seek to find a minimal variance estimator of *X*. Note that in the upper panel points were individually shifted in the *X* dimension to modify the variance of *X* while points in the lower panel were shifted by the same amount and direction in the *X* dimension to modify bias. Points have the same *Y* value across all plots.

However, coherence is commonly estimated using the multitaper method^9^, which is a clever technique that projects data onto multiple orthogonal functions (tapers) in order to produce multiple independent power spectral estimates. The key advantage of this approach is that it provides a lower-variance estimate^10^ of frequency coupling, which is critical in estimating Pearson correlation coefficients (Fig. 1) between such coupling and task-relevant phenomena such as task performance. Specifically, the maximum measurable correlation coefficient between two quantities is inversely related to the amount of estimation variance^11–15^. In this work, we consider correlations between frequency coupling and the particular phenomena of visual task performance. This is motivated by the fact that frequency-domain activity has been shown to be relevant to task performance^16–19^. Therefore, the first key objective of this work is to develop a multitaper estimation technique for MIF to reduce estimator variance, and a toolbox for this estimator has been made available on GitHub (https://github.com/jy46/MIF-Toolbox, 10.5281/zenodo.3879802). The previous work^5^ did not take advantage of the multitaper method, and its introduction will provide more robust estimates of correlations between frequency coupling and task-relevant phenomena. In particular, we are able to use multitaper MIF to more precisely measure the association between frequency coupling and visual task performance.

We also explore how this new multitaper estimator of MIF and coherence differ for simulated non-Gaussian Processes (non-GPs), since coherence can only fully describe GPs. In fact, prior work^5^ provided an analytic transformation from coherence to MIF for such GPs. Since coherence only relies on the second-order statistics of the two processes being analyzed for frequency coupling, one naturally wonders if this transformation will underestimate coupling in the case of non-GPs, which possess higher than second-order characteristics. In order to address this question, we perform simulations and demonstrate that the transformation indeed provides an underestimate of the MIF value, providing further evidence of MIF’s ability to capture higher than second-order relationships and suggesting that coherence is missing information for non-GPs.

## Methods

### Maximizing correlations

Frequency coupling is often correlated with task-relevant phenomena, such as task performance. A key consideration that is often overlooked is the variance of one’s estimator for *X* or *Y* when computing the Pearson correlation coefficient between them. By considering the error or variance present in estimating *X* or *Y*, it can be shown that the measured correlation ${\hat{\rho }}$ is guaranteed to be lower than the true correlation $\rho$ and that the estimation variance is inversely related to this measured correlation^11–15^.

To put this into context, imagine the case where frequency coupling *X* and a subject’s task accuracy *Y* are perfectly correlated ($\rho =1$). If *X* and *Y* could be measured with zero error/variance, then the measured correlation would accordingly be 1 (Fig. 1, top-left). However, if we simply consider the presence of error/variance in estimating *X* (Fig. 1, top-middle and top-right), then the estimated correlation will decrease. Intuitively, we know that a higher variance estimator of *X* will produce points that are more likely to be off of the line existing in the *XY*-plane that represents the underlying perfectly linear relationship between *X* and *Y*, reducing the measured correlation.

We further note that the bias of one’s estimate, i.e. a general offset in estimates, does not effect the measured correlation (Fig. 1, bottom panel) since the data is centered via mean subtraction when computing the Pearson correlation coefficient. Therefore, a chief priority when considering correlations between frequency coupling and task-relevant phenomena is to reduce variance in frequency coupling estimation so that the measurable correlation is increased and thereby more accurately assessed.

### Coherence

Coherence is a frequency coupling metric based on the correlation between two time series in the frequency domain that is a comprehensive measure of statistical dependence only in the case of linearly-related GPs^5,20^. Consider a random process *X*(*t*) with *t* taking on discrete values $t_1$, $t_{2}$, ..., $t_n$. If $[X(t_1)$, $X(t_{2})$, ..., $X(t_n)]$ follows a multivariate Gaussian distribution, then it has a covariance structure $\Sigma$ and is a GP. If we have a similarly defined process *Y*(*t*), the coherence between *X*(*t*) and *Y*(*t*) is defined mathematically as^20,21^:

1 $$\begin{aligned} C_{XY}(f) = \frac{|S_{XY} (f)|^2}{S_{X} (f)S_{Y} (f)}, \end{aligned}$$

where $S_{X}(f)$ and $S_{Y}(f)$ are the power spectral densities of stochastic processes *X*(*t*) and *Y*(*t*), respectively, while $S_{XY}(f)$ is the cross power spectral density between *X*(*t*) and *Y*(*t*). Note that this definition (1) has the advantage of being real valued^20^. For frequencies for which the stochastic processes *X*(*t*) and *Y*(*t*) are uncorrelated, the coherence is 0, while for perfectly correlated frequencies it will be 1^20^.

**Figure 2:**
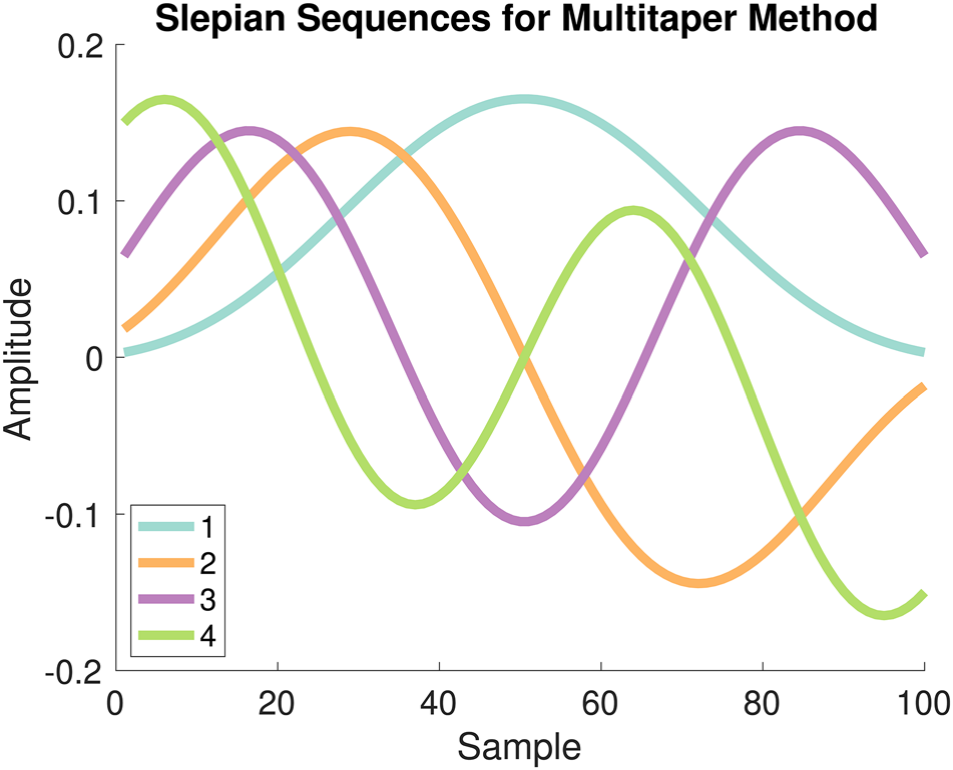
Example tapers^22^ used in the multitaper technique^9^. Data windows would be multiplied element-wise by the tapers, and then input into the FFT in order to produce independent spectral estimates.

In order to estimate the coherence between two random processes *X*(*t*) and *Y*(*t*), one takes non-overlapping windows (trials) of sample paths of both processes and then point-wise multiplies them separately by each of the orthogonal taper functions^9,22^ (Fig. 2). The FFT and complex conjugate of the FFT of these windows are then multiplied to produce estimates of $S_{X}(f)$ and $S_{Y}(f)$ for each trial and taper. Similarly, the FFT of the windows corresponding to *X*(*t*) is multiplied with the complex conjugate of the windows corresponding to Y(t) to provide estimates of $S_{XY}(f)$. All estimates are averaged across trials and tapers to produce ${\hat{S}}_{X}$, ${\hat{S}}_{Y}$, and ${\hat{S}}_{XY}$, which are finally plugged into (1). This multitaper approach reduces estimation variance^10^, and thereby increases the measurable correlation (Fig. 1) between coherence and task-relevant phenomena (as per the previous section)^11–15^. In the next section, we will describe how we take advantage of this multitaper approach for MI in frequency estimation.

### MI in frequency (MIF)

Given that neural signals are almost always non-Gaussian^5^ one must consider non-GPs involved in higher-order relationships, which our powerful MIF estimator is well-suited to capture. In contrast to measures such as coherence^6^ and traditional cross-frequency coupling metrics^23^, mutual information (MI) measures statistical dependence for linear and nonlinear relationships. Importantly, it makes no modeling assumptions. Considering two continuous random variables X and Y, the MI between them is defined as^24^:

2 $$\begin{aligned} I(X;Y)= h(X)+ h(Y)- h(X,Y), \end{aligned}$$

where $h(X) = -\int _S p(x)\log p(x) dx$^24^, i.e., the differential entropy of *X*, and *p*(*x*) is the probability density associated with *X* and *S* denotes the support of *X* where $p(x)>0$. We note that the joint differential entropy *h*(*X*, *Y*) is similarly defined: $h(X,Y) = -\int _S p(x,y)\log p(x,y) dxdy$^24^. In terms of intuition, *h*(*X*) is a measure of the uncertainty of *X* and therefore MI can be thought of as the information that *X* and *Y* provide about each other. Probabilistically, it is a measure of dependence where an MI value of 0 represents full independence between *X* and *Y* and any value greater than 0 represents dependence between *X* and *Y*. Note that the MI between two continuous random variables is a non-negative quantity, and is unbounded.

However, since our analysis is focused on time series data, specifically local field potentials (LFPs), dependency exists between time samples and therefore random processes *X*(*t*) and *Y*(*t*) must be considered rather than the aforementioned random variables. A recent formulation^5^ inspired by previous work^8^ showed how MI can be applied to the frequency domain representations of *X*(*t*) and *Y*(*t*) by using Cramér’s representation of a random process^25,26^. The following is the corresponding time and frequency relationship for *X*(*t*), which follows for *Y*(*t*), as well as the explicit frequency domain representation of *X*(*t*)^5,25,26^:

3 $$\begin{aligned} X(t) = \int _{-\infty }^{\infty }e^{j2\pi f t}d{\tilde{X}}(f), \quad{\tilde{X}}(f) = {\tilde{X}}_R(f) + j{\tilde{X}}_I(f), \end{aligned}$$

where $d{\tilde{X}}(f)$ is referred to as an increment and the integral displayed is a Fourier-Stieltjes integral. Note that we slightly redefine $d{\tilde{X}}(f_i)$ to now refer to the random vector $[d{\tilde{X}}_R(f_i),d{\tilde{X}}_I(f_i)]$, where $d{\tilde{X}}_R(f_i)$ and $d{\tilde{X}}_I(f_i)$ are the real and imaginary components of $d{\tilde{X}}(f_i)$, respectively. Note that this is done similarly for $d{\tilde{Y}}(f_i)$. MI in Frequency (MIF) was formulated^5^ using the increments $d{\tilde{X}}(f_i)$ and $d{\tilde{Y}}(f_j)$ of ${\tilde{X}}(f)$ and ${\tilde{Y}}(f)$ at frequencies $f_i$ and $f_j$. Therefore MIF, denoted $MI(\cdot ,\cdot )$, can be defined as^5^:

4 $$\begin{aligned} MI(f_i,f_j) = I(d{\tilde{X}}(f_i);d{\tilde{Y}}(f_j)), \end{aligned}$$

which can also be defined in terms of entropy:

5 $$\begin{aligned} MI(f_i,f_j) = h(d{\tilde{X}}(f_i)) + h(d{\tilde{Y}}(f_j)) - h(d{\tilde{X}}(f_i),d{\tilde{Y}}(f_j)). \end{aligned}$$

Since MIF can be computed between frequency $f_i$ of $d{\tilde{X}}$ and a different frequency $f_j$ of $d{\tilde{Y}}$, this results in an MIF matrix indexed by the possible frequency pairings. This highlights the power of MIF to quantify nonlinear relationships across frequencies, which is fundamentally different from the restriction of coherence to same-frequency interactions as made clear by the single frequency indexing in (1). Finally, we mention that coherence (1) has a direct relationship to MIF for linear GPs^5^:

6 $$\begin{aligned} MI(f,f)=-log(1-C_{XY}(f)). \end{aligned}$$

**Figure 3:**
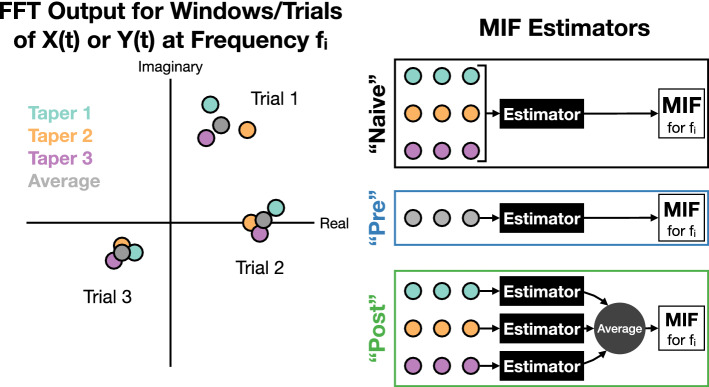
Three possible approaches for multitaper MIF estimation. The plot on the left depicts how the FFT output for a particular frequency $f_i$ of windows/trials of *X*(*t*) or *Y*(*t*) could look with multiple tapers applied, and “Average” refers to the data produced by averaging across samples produced by the different tapers. MIF can be estimated from this data in the three different ways depicted on the right, where estimator refers to the *k*-nn MI estimator^28^.

In order to estimate MI in the frequency domain between two random processes *X*(*t*) and *Y*(*t*), we augmented the procedure described in^5^ with the multitaper approach^9^. One begins by taking non-overlapping windows of sample paths of both processes, and independence between windows is assumed. For example, one could use windows of recordings at the same relative time point (e.g. stimulus onset) across trials of an experiment, meaning that windows in that case will correspond to trials. In a different scenario, such as the resting state where no stimuli are being introduced, one could reasonably assume that recordings are stationary and therefore consecutive non-overlapping windows of each channel could be used without regard to any notion of “trials”. The former approach describes the process that we will use in our analyses and therefore our wording describes that context, however this process is easily applicable to the latter scenario by considering trials to just mean windows. In either case, the acquired windows are then point-wise multiplied separately by each of the orthogonal taper functions^9,22^ (Fig. 2). The Fourier transform of each of these is computed at a given frequency $f_i$, providing samples for estimating the increments $d{\tilde{X}}(f_i)$ and $d{\tilde{Y}}(f_i)$^27^. We mention that the Fourier transform of these windows will yield two-dimensional samples of $d{\tilde{X}}(f_i)$ and $d{\tilde{Y}}(f_i)$ for each frequency $f_i$, with one dimension corresponding to the real component, i.e. $d{\tilde{X}}_R(f_i)$ and $d{\tilde{Y}}_R(f_i)$, and the other dimension corresponding to the imaginary component, i.e. $d{\tilde{X}}_I(f_i)$ and $d{\tilde{Y}}_I(f_i)$. These samples which are specific to one frequency can be seen in our toy example in Fig. 3, where each window/trial provides a complex sample for each taper. Technically, there would be two plots: one for samples of $d{\tilde{X}}(f_i)$, and one for samples of $d{\tilde{Y}}(f_i)$.

Samples of $d{\tilde{X}}(f_i)$ and $d{\tilde{Y}}(f_i)$ across trials for the same frequency $f_i$ are then plugged into a *k*-nearest neighbors (*k*-nn) entropy estimator^28–31^ from which MIF can then be measured^5^. Since variance reduction is our focus, *k* is chosen to be half the number of samples as per the recommendation of prior work^28^. However, one must consider how to handle the fact that these samples were acquired by different tapers. We can in fact implement three different multitaper approaches (Fig. 3). We can naively input all samples across tapers into the estimator together, or we can pre-average samples across tapers in the complex plane before inputting them into the estimator, or finally we can post-average MIF estimates across tapers by computing independent MIF estimates specific to each taper and then averaging them. We note that for coherence the naive and pre-averaging approaches turn out to be equivalent to each other and to the traditional method of estimating coherence. The post-averaging approach to coherence is not investigated since the primary focus of this paper is to develop a better MIF estimator and compare it with the commonly used approach to coherence. We further know that a post-averaged coherence would still fail to capture features detected by MIF.

In the results section we will detail the performance of the “pre” and “post” estimators in terms of variance, and also compare them with the more traditional approach of using Hamming windows. This will reveal that often post is the lowest-variance estimator and therefore is the best estimator for correlating frequency coupling with task-relevant phenomena (Fig. 1). We found that the “naive” approach yields the highest variance, and therefore we do not focus on it in our results. Again, the lower the estimation variance, the higher the maximum correlation that we can measure^11–15^.

## Results

The following sections detail the key components of our results: (1) determination of the multitaper MIF estimator with low variance and the best performance for correlation analyses, (2) analysis of how much more information MIF captures compared to coherence in simulations, and (3) use of our MIF estimator and coherence to explore correlations between frequency coupling and visual task learning.

**Figure 4:**
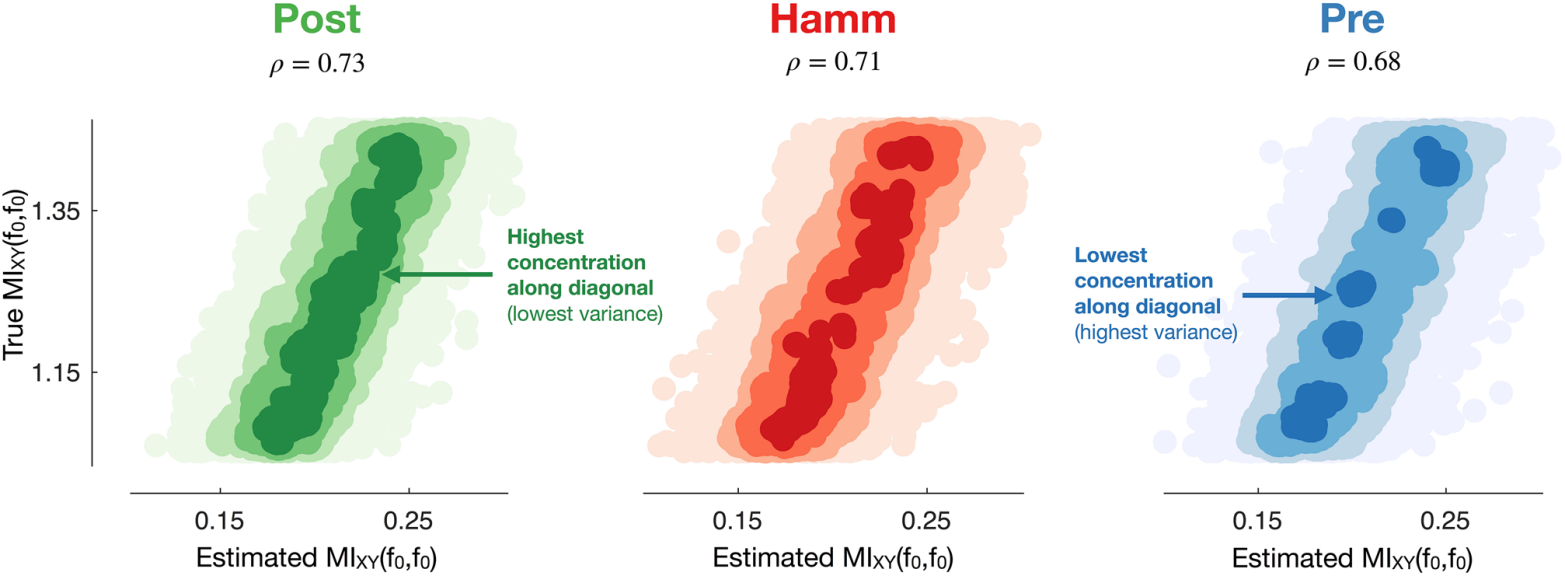
Density scatter plots^32^ comparing Pearson correlation coefficients $\rho$ between true and estimated MIF values for frequency $f_0$ for different MIF estimator types applied to samples from the random sinusoid model (7–9). A perfect estimator would have a correlation of 1 between its estimates and the true values, meaning that post is the best estimator since it has the highest correlation. Visually it has the highest density of estimates along the diagonal, which is an indicator of lower estimation variance and higher correlation between estimates and true values.

### MIF estimator comparison

For comparing the variance of the proposed multitaper MIF estimators and determination of how well their estimates would correlate with task-relevant phenomena, we utilized a random sinusoid model similar to what was used in prior work^5^:

7 $$\begin{aligned} X(t)&= Acos(2\pi f_0t+\Theta ) \end{aligned}$$

8 $$\begin{aligned} W(t)&= Bcos(2\pi f_0t+\Phi ) \end{aligned}$$

9 $$\begin{aligned} Y(t)&= X(t)+W(t) \end{aligned}$$

where $A \sim Rayleigh (1)$, $B \sim Rayleigh (\sigma _B)$, $\Theta \sim Uni\mathit{f}orm (0,2\pi )$, and $\Phi \sim Uni\mathit{f}orm (0,2\pi )$. We note that *X*(*t*), *W*(*t*), and *Y*(*t*) are GPs, and therefore the true MIF between *X*(*t*) and *Y*(*t*) for $f_0$ is known^5^ to be:

10 $$\begin{aligned} MI_{XY}(f_0,f_0) = log\left( 1+\frac{S_{X}(f_0)}{S_{W}(f_0)}\right) = log\left( 1+\frac{1}{\sigma _B^2} \right) . \end{aligned}$$

For any other frequency besides $f_0$, the MIF between *X*(*t*) and *Y*(*t*) will be zero.

A perfect estimator with no estimation variance would have a Pearson correlation coefficient of 1 between its estimates and true $MI_{XY}(f_0,f_0)$ values (see Fig. 1 for intuition), and therefore we determine which estimator is best by exploring which has the highest correlation with true MI in frequency values. 100 sample paths of the sinusoids (7–9) were generated for a fixed $\sigma _B$, meaning the true MIF value was known (10). This corresponds to 100 trials or windows of experimental data, a common regime for researchers. We note that the sample path or window length is proportional to the frequency resolution one can achieve, and therefore one would have to consider trade-offs between frequency resolution and the number of sample paths or windows chosen for real data. Since this is an example and since only the number of sample paths corresponds to the number of data points used to estimate MIF, the sample path length and $f_0$ can be chosen arbitrarily and have no effect on this example analysis. MIF was estimated from these sample paths using the different taper approaches with the *k*-nn MI estimator^28^, and this process was performed 10,000 times for a specific range of $\sigma _B$ values in order to determine the correlation between estimates and true values. Figure 4 displays the results of these simulations and shows that the post estimator is the best estimator because it has the highest correlation with true MI in frequency values. Qualitatively, the estimation variance of post also appears to be the lowest since it visually has the highest density of points along the diagonal.

**Figure 5:**
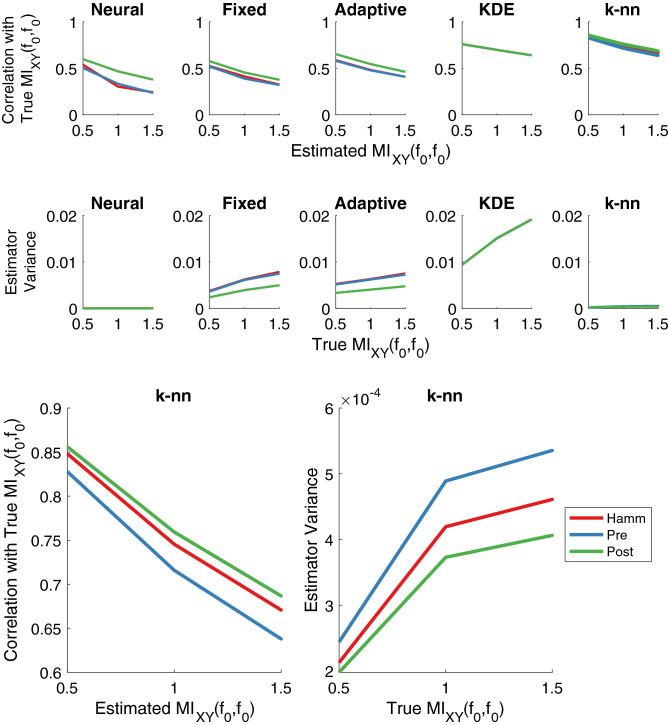
Top row: performance of MIF estimation approaches measured by the correlation between estimated and true MIF values for frequency $f_0$ (7–9). *k*-nn with the post multitaper approach performs best (highest correlation). Each point is the correlation between 1e4 MIF estimates (1e3 for KDE and the neural network because of infeasible computation time) using 100 sample paths and a range of true MIF values, the center of which is used as the *x* value. Ranges spanned $\pm 0.2$ around the center. Second row: estimator variance for different true MIF values. Except for the neural network and Kernel Density Estimation (KDE), post has the lowest variance across all approaches which corresponds to it having the highest performance across windowing methods. Pre exhibits slightly lower variance than post for the neural network and KDE displays no differentiation for windowing methods. Sample sizes same as top row. Bottom: enlargement of *k*-nn correlation and variance plots.

To put this into context, we could consider the scenario where a task-relevant phenomena is perfectly correlated with frequency coupling. For example, in the final section of our results, we analyze how macaque task performance correlates with coupling. In such cases, the variance of the estimation technique limits the maximum correlation that can be measured between such task-relevant phenomena and coupling^11–15^, and therefore our result shows that the post estimator should be used.

The top row of plots in Fig. 5 expands upon Fig. 4 by considering a variety of separate ranges for true MI values and displays the correlations between those values and the estimates produced not only by *k*-nn but also by four other MI estimation techniques. Specifically, we considered neural network^33^, fixed-bin histogram^34^, adaptive-bin histogram^35,36^, and Kernel Density Estimation (KDE)^5,37,38^ approaches to MI estimation. We utilized two bins for the fixed-bin histogram and three bins for the adaptive-bin histogram approaches, as these generally produced the highest correlations between MI estimates and true values across different bin numbers, of which only lower bin numbers were considered since these will produce lower estimation variance. The bandwidth of the KDE kernel was chosen via smoothed cross-validation^39^. Overall, we found that the *k*-nn approach combined with the post multitaper method performed best in terms of producing the highest correlations between estimates and true MI values (top right plot in Fig. 5). This is consistent with the previous figure, further confirming post *k*-nn is the best estimator for frequency coupling in correlation studies. The decrease in performance associated with higher true MI values exhibited across all of the estimators tested has been noted previously to be an issue in MI estimation^40^. Histogram and *k*-nn methods used 10,000 MIF points estimated from 100 sample paths for correlation values, while KDE and the neural network approach used 1000 MIF points estimated from 100 sample paths because of infeasible computation time.

KDE did not exhibit any differences across windowing approaches, while for every other MI estimator our post approach produced the best performance out of the windowing methods. KDE’s insensitivity to the windowing methods is likely due to the small grid size used in its implementation (“ks” package for R^41^), which cannot be increased without becoming computationally infeasible for this analysis. For the neural network approach, which is called Classifier based Conditional Mutual Information (CCMI), we used code available on GitHub (https://github.com/sudiptodip15/CCMI)^33^, and for the adaptive-bin histogram method we used the code provided with a previous work^36^.

The second row of plots in Fig. 5 quantitatively explore the estimator variance that was visually observed in Fig. 4. This was done by generating 100 sample paths (7–9) and estimating MIF, then repeating this 10,000 times (1000 for CCMI and KDE because of infeasible computation time) in total for each true MI value. Finally, the variance of the estimates for each true MI value was computed and plotted. For the fixed-bin histogram, adaptive-bin histogram, and *k*-nn approaches there was a general inverse relationship between estimator variance and estimator performance (as measured by the correlation between estimates and true values), where post produced the best performance and lowest variance among the windowing approaches within each estimation technique. KDE exhibited no distinction between variances for the three windowing approaches, which is consistent with it not exhibiting any difference in terms of performance across the windowing approaches and is undoubtedly due to the grid size issue mentioned previously. Although not visible in the figure, using the pre windowing approach for CCMI actually produces lower variance than the post approach. It is unclear why this is the case, however we note that variances for CCMI are remarkably low (on the order of $10^{-5}$ and smaller) which causes one to wonder about precision issues for variance measurement.

The variances in the second row of plots in Fig. 5 should not be compared across MI estimation methods. Although it is one of the primary emphases of our work that reducing estimator variance is key to producing better estimators for correlation analyses, variance by itself does not tell one anything about an estimator’s performance. To provide intuition, we consider an estimator that produces a constant value independent of the true MI. The variance of this estimator will be zero, but to its detriment the correlation between its estimates and true values will also always be zero. Therefore, variances should generally only be compared across windowing approaches within an MI estimator type and should always be considered in combination with performance as measured by correlations between estimates and true values.

**Figure 6:**
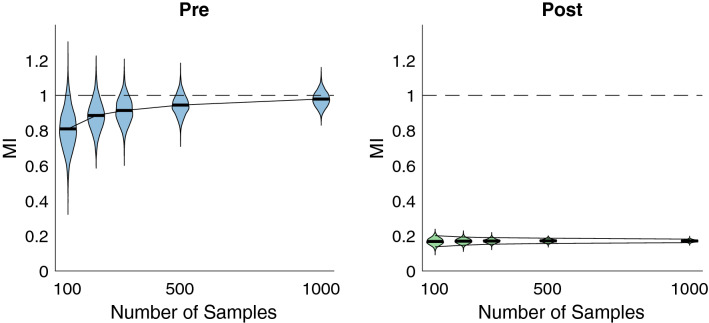
Analysis of asymptotic properties of pre and post approaches with the *k*-nn estimator. Left: violin plots^42^ display the distribution of MIF estimates as a function of sample size in relation to the true MIF value indicated by the dashed line. 1e3 estimates comprise each distribution. The trendline intersects the mean of each distribution in order to indicate decreasing bias with increasing number of samples, while the narrowing of the distributions indicate decreased variance with increased sample size. Right: same analysis performed for the post estimator, which exhibits poor bias but considerably smaller variance than the pre estimator. The decrease of this variance with sample size is highlighted by the two trendlines following the 5th and 95th percentiles of each distribution.

We also analyzed the asymptotic properties of the pre and post *k*-nn estimators in Fig. 6 by considering different sample sizes with 1000 estimates used for each sample size. With *k* set to 3 for the pre estimator in order to emphasize its focus on bias reduction, the left plot in Fig. 6 demonstrates the strong degree to which bias is removed with increasing sample size. In contrast, with *k* set to half the sample size for the post estimator in order to emphasize its focus on variance reduction, the right plot in Fig. 6 displays how this variance reduces with sample size and that it is considerably lower than that of the pre approach on the left. It is clear from this figure that variance is reduced with increasing sample size for either approach, implying that once a certain sample size threshold is reached that the difference in variances between the two approaches may become negligible. For the next section where we compare the level of information captured by MIF versus coherence, the pre estimator is ideal because of its remarkable bias reduction and respectable variance reduction.

### MIF and coherence: simulations

To compare the information captured by MIF and coherence, we started by computing both of these quantities on simulations of the GP model (7–9) introduced in the previous subsection with $\sigma _B=1$. However, in order to evaluate asymptotic behavior, we increased the number of generated sample paths from 100 to 10,000 and utilized the pre estimator because of its lower bias (Fig. 6) which is useful for this particular analysis. This is because we are comparing MIF and coherence values to determine the level of information captured by each metric, making lower bias desirable in this case. However, we note that the information advantage shown for pre MIF will also be fully present in post MIF, because both will still measure statistical dependence while coherence will only measure correlations between frequency components. We further note that because it was computationally infeasible to perform the analysis in Fig. 6 for a sample size of 10,000 with 1000 iterations, the estimates in this section were performed 100 times in order to provide error bars around each estimate to still give a sense of the spread and to therefore indicate where MI and coherence truly differ.

**Figure 7:**
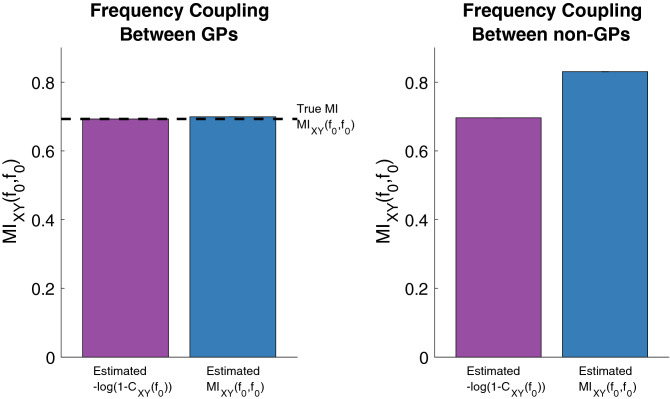
Comparison of frequency coupling estimated by MIF and coherence (C) between GPs and non-GPs, with the estimated coherence being transformed to MIF (6) and accordingly labeled “$-log(1-C_{XY}(f_0))$”. Left: both MIF and the transformed coherence capture all information about the coupling between GPs, as evidenced by the $-log(1-C_{XY}(f_0))$ value being basically identical to the estimated MIF. 1e4 samples were used for each estimate, with 100 iterations performed in order to provide variance errorbars for each estimate (which are too small to be easily visible). Right: the $-log(1-C_{XY}(f_0))$ transformation underestimates the MIF value, implying that coherence is not capturing all of the information about the coupling between non-GPs.

The results of this comparison are shown in the left hand side in Fig. 7. Because the simulations utilize GPs, we can compute the transformation from coherence to MIF by applying the previously discussed formula ($MI_{XY}(f,f)=-log(1-C_{XY}(f))$^5^) to the estimated coherence. Including this calculation in Fig. 7 as the first bar confirms that this equation accurately models the relationship between MIF and coherence for GPs since it takes on a similar value to that of MIF (first bar). In other words, it would seem coherence and MIF capture the same level of information for simulated GPs.

However, a simple change to the distribution of $A$ and $B$ from Rayleigh to uniform in the previous model, i.e. *A*, $B\sim$ *Uniform*$(-0.5, 0.5)$, causes this model to be a non-GP. For example, the 4th moment (kurtosis) can be computed analytically to be 2.7. Since the kurtosis of a Gaussian process is 3, we can say that this model is now a non-GP.

The plot on the right hand side in Fig. 7 displays the results of applying multitaper MIF and the transformed coherence to this new model. For this non-GP model, using the coherence-to-MIF equation underestimates the MIF that is estimated from realizations of the simulation. This highlights the gap in coupling measurement, i.e. the lack of information captured by coherence, that could occur if one is only considering second-order statistics rather than full statistical dependence.

### MIF and coherence: visual cortical data

**Figure 8:**
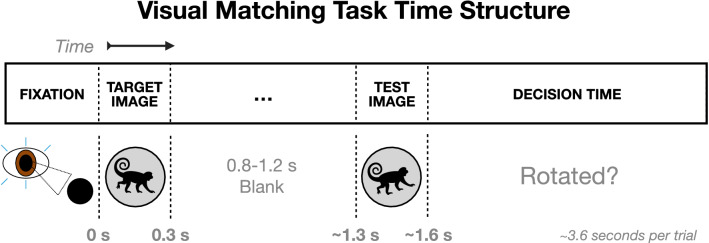
Time structure of each trial of the visual delayed matching-to-sample task learned by two rhesus macaques^16^. Monkeys fixated for 0.5 s, then two images were displayed in the periphery of their visual field for 0.3 s each, with a 0.8–1.2 s blank period in between. Macaques were then given 1.5 s to respond whether or not the identical images had matching orientations.

Having developed the lower variance post multitaper MIF estimator, we then explore how it performs compared to coherence on visual cortical recordings. In particular, we analyzed local field potential (LFP) recordings from the activity of neurons in mid-level visual cortex (area V4) while two monkeys learned a simple image rotation task^16^ as portrayed in Fig. 8. Rhesus macaques were presented with two stimuli, each displayed for 300 ms with a 0.8–1.2 s blank period in between. The first stimulus was a gray scale circular image of a natural scene, while the second was a potentially rotated (up to $20^{\circ }$) version of the first. Monkeys then decided if image orientations were a *match* or *non-match*, and received a juice reward for responding correctly within 1.5 s after the offset of the second image. Therefore, each trial was approximately 3.6 s in total. For each day, the image shown in each trial was the same, however different images were shown across days. Accordingly, on learning days monkeys were presented with novel imagery, whereas on non-learning days monkeys were presented with previously seen imagery. The analyses of this work focus on learning days, since larger changes in task accuracy allow for a correlation analysis between accuracy and coupling measured by coherence or MIF.

LFPs for one monkey were recorded using two 16-channel Plexon U-Probes with electrodes $100\,\upmu \hbox {m}$ apart while for the other monkey recordings were from pairs of MPI microelectrodes^16^. For MIF calculations, we directly used the channels of the microelectrodes for the second monkey while using averages across channels within probes for the first monkey because of channel proximity.

**Figure 9:**
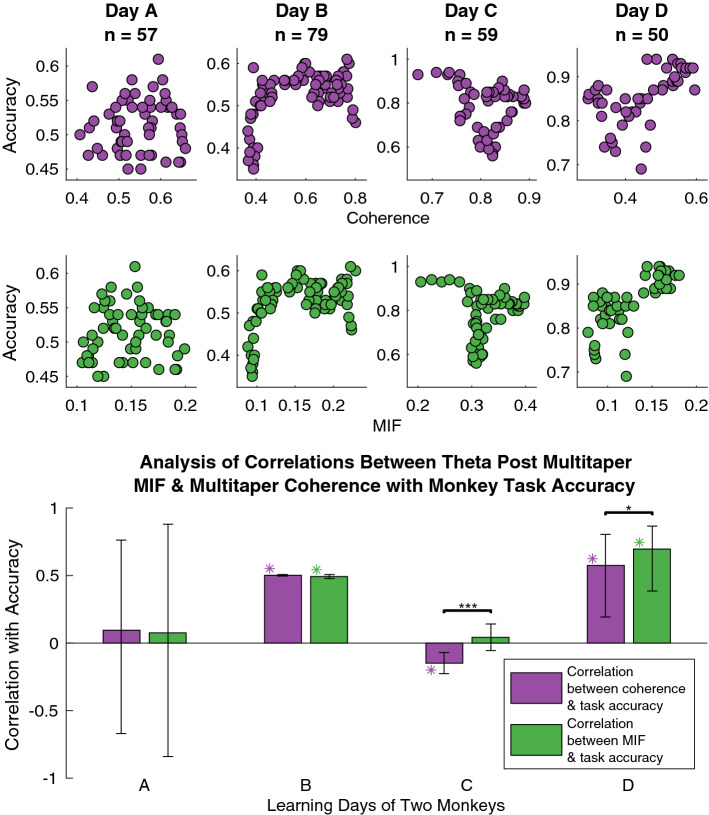
Top: scatter plots of average monkey task accuracy against coherence/MIF. Each point is comprised of 100 trials, and *n* indicates the number of points in each plot. Coherence and post MIF exhibit minor differences for each day. Bottom: correlations between coherence/MIF and accuracy based on scatter plot data are compared. Similar to the scatter plots, coherence and post MIF tend to exhibit minor differences in their correlations with accuracy for this data set. We utilized a software package^43^ to estimate confidence intervals for correlation estimates, where a purple/green “$$*$$” designates a significant Pearson correlation coefficient *R* for which the 95% confidence interval (calculated to account for autocorrelation induced by sliding overlapping windows) excludes zero. For the black “$$*$$”^44^ $$p=0.0268$$ and for “$$***$$” $$p=0.0002$$ regarding the significance of correlation differences^45,46^. Differences on days C and D are statistically significant, but because correlations are low on day C and both are positive on day D we consider coherence and MIF to mostly agree for this particular data.

Our analysis focuses on the theta band (4–8 Hz) because of inspiration from previous analyses. Specifically, prior analysis^16^ of this particular data set found heightened theta band (4–8 Hz) spike-field coherence associated with learning during each stimulus presentation, and other work on a similar task found interesting theta (3–9 Hz) coupling and performance results during the delay period^47^. The former analysis^16^ focused on spike-field coherence, i.e. coherence between spike timings and LFPs, while we analyze coupling instead between LFPs, both in terms of coherence and MIF. The latter analysis^47^ was categorically different from our findings, since its timing suggests an association with short-term memory. In contrast, we analyze phenomena taking place approximately during the latter part of image two presentation continuing into the beginning of the decision period.

In particular, we analyzed theta (4–8 Hz) coupling between V4 LFPs in the time window starting at 1.6 s and ending at 2 s from the first stimulus onset. This window takes place approximately during the latter part of image two presentation continuing into the beginning of the decision period. We used 300 ms windows starting at 1.6, 1.65, and 1.7 s to get three different estimates of coherence and MIF within the theta band for the first 100 trials of a given experimental day for a given monkey. 300 ms was chosen since that is the length of stimulus presentation, and 100 trials were utilized to provide a reasonable coherence/MIF estimate. These three estimates were averaged to produce one estimate of coherence and one estimate of MIF for that window of trials. Then, we slid the window of 100 trials by ten trials and computed new estimates, and continued this until reaching the end of the collection of trials for the given day. A sliding window of trials was implemented in order to provide more data points in correlating frequency coupling with accuracy, and we accordingly utilized a software package^43^ to estimate confidence intervals that account for the dependency between windows that this introduces. This process produced a series of coherence estimates and a series of MIF estimates indexed by the particular window of trials. Similar to a prior significance testing approach^48^, coherence and MIF values were significance tested by comparison with a threshold equal to the 95th percentile of the maximum distribution, which was estimated with 5000 samples each resulting from taking the maximum coherence/MIF value across frequency for a given permutation of the set of the trial windows of one of the two LFPs. Monkey task accuracy was also computed for the (unpermuted) windows of trials, and then correlated with the significance tested coherence and MIF estimates. This was done for two monkeys with 2 days of data each, producing four correlations between task accuracy and coherence, as well as between task accuracy and MIF, which are displayed in Fig. 9.

In Fig. 9 each pair of bars is a result for the same learning day, with days A and B coming from one monkey and days C and D coming from a second monkey. Both post MIF and coherence in the theta band (4–8 Hz) exhibit similar behavior for this data. Note that we utilized a software package^43^ to estimate confidence intervals for the correlation estimates in Fig. 9, where a purple/green “$$*$$” designates a significant Pearson correlation coefficient *R* for which the 95% confidence interval excludes zero. The confidence intervals account for autocorrelation induced by the sliding window approach via use of a block bootstrap technique^43,49–52^. Although unspecified by the software, the relevant papers^43,49^ imply that 2000 bootstrap samples were used, along with 1000 resamplings of each of those bootstrap samples in order to improve confidence interval accuracy^43^. Furthermore, a black “$$*$$”^44^ indicates $$p\le 0.05$$ and “$$***$$” indicates $$p\le 0.001$$ for the significance of correlation differences^45,46^ via a two-tailed Z-test which accounted for the fact that both correlations have accuracy as a common variable. Although days C and D show statistically significant differences, we consider coherence and MIF to be showing generally the same findings for this particular data set because the correlations on day C are very low and both correlations are positive on day D.

## Discussion

Interestingly, the post approach to multitaper MIF estimation provides very low variance (Fig. 5) when using *k*-nn, which suggests that averaging MIF estimates across tapers is the key to reducing variability in estimates for *k*-nn. As to why post produces lower variance than pre, we consider a high level hypothesis where an MIF estimator is taken to be a highly nonlinear and nonconvex function that receives complex samples as input and produces an MIF estimate as output. The nonlinearity and nonconvexity of this function will cause it to be rife with hills and valleys. Therefore, even though the spectral estimates in the pre case have been averaged, there are no strong guarantees on what output the MIF function will give for these spectral inputs. In fact, these inputs could cause the function to take a value at the peak of a hill or at the floor of a valley. By contrast, the post estimator is able to average across function outputs, meaning that this landscape ridden with hills and valleys is effectively low-passed and therefore the estimator variance is reduced. In accordance with the notion of the bias-variance trade-off, this variance reduction comes at a potential cost: the post approach has higher bias than the pre approach, and also has a higher bias than the traditional Hamming approach. However, the primary focus of this work was on changes in frequency coupling that correlate with task phenomena rather than the absolute values of frequency coupling, making variance rather than bias the priority. When absolute values are important, such as in our comparison between MIF and coherence on simulated data, the pre approach proves useful.

In comparing coherence and MIF for simulations of GPs and non-GPs, it is remarkable how a change from Rayleigh to uniform distributions appeared to cause information to be missed by coherence. This is concerning since sinusoidal waveforms with uniformly distributed amplitudes seem biologically plausible, yet the commonly used metric of coherence will not entirely capture relationships between such signals. In contrast, MIF will capture the full statistical dependence between these signals. Further models should be simulated to see how this phenomena translates across them. Additionally, we recognize that the statistical power (sensitivity) for detecting an effect could defer between coherence and MIF, and therefore we suggest this as another important avenue for future work.

Our development of a lower-variance *k*-nn estimator for MIF was critical in accurately measuring correlations between frequency coupling and visual task performance, since higher variance reduces the maximum measurable correlation^11–15^. For our particular data set, coherence and MIF behaved mostly similarly. However, theoretically, we know that MIF captures full statistical dependence while coherence is limited to second-order statistics. Therefore, future work should demonstrate this fact empirically with a suitable data set that exhibits stronger deviations from Gaussianity.

Additionally, we briefly consider interpretation of the results found from applying MIF to the visual cortical data. If the MIF values displayed in Fig. 9 are in fact all accurate, it could possibly be concluded that theta MIF is associated with macaques’ general state of alertness. As pointed out previously^16^, heightened LFP power^17,18^ within the gamma band is one example of a quantity among others that has correlated with a higher state of arousal. However, significance testing makes it clear that theta coupling measured by MIF is considerably more likely to be capturing phenomena that is more specific than general arousal, since one day of each monkey exhibits significant positive correlations between theta MIF and accuracy, while the other day of each monkey has no significant correlation.

In conclusion, we developed a powerful approach to performing estimation of MIF for maximizing measurable correlations with task-relevant phenomena in the form of the “post” *k*-nn estimator, which was associated with lower estimation variance than other *k*-nn methods. Furthermore, simulations revealed MIF’s superiority in general over coherence to fully capture frequency coupling by being more informative. We found that coherence and MIF mostly agreed when applied to our particular visual cortical LFP data set, however we expect that in many cases MIF will outperform coherence empirically and future work should focus on this application. Therefore we provide neuroscientists with a robust way to measure frequency coupling that is ideal for correlating such coupling in neural data with task-relevant phenomena.

## Data Availability

We provide access to our MIF toolbox here: https://github.com/jy46/MIF-Toolbox (10.5281/zenodo.3879802). The datasets generated during and/or analysed during the current study are available from the corresponding author on reasonable request.
